# Duration of exclusive breastfeeding is associated with differences in infants’ brain responses to emotional body expressions

**DOI:** 10.3389/fnbeh.2014.00459

**Published:** 2015-01-22

**Authors:** Kathleen M. Krol, Purva Rajhans, Manuela Missana, Tobias Grossmann

**Affiliations:** ^1^Early Social Development Group, Max Planck Institute for Human Cognitive and Brain SciencesLeipzig, Germany; ^2^Department of Psychology, University of Virgina, CharlottesvilleVA, USA

**Keywords:** breastfeeding, emotion, infants, ERP, body expressions

## Abstract

Much research has recognized the general importance of maternal behavior in the early development and programing of the mammalian offspring’s brain. Exclusive breastfeeding (EBF) duration, the amount of time in which breastfed meals are the only source of sustenance, plays a prominent role in promoting healthy brain and cognitive development in human children. However, surprisingly little is known about the influence of breastfeeding on social and emotional development in infancy. In the current study, we examined whether and how the duration of EBF impacts the neural processing of emotional signals by measuring electro-cortical responses to body expressions in 8-month-old infants. Our analyses revealed that infants with high EBF experience show a significantly greater neural sensitivity to happy body expressions than those with low EBF experience. Moreover, regression analyses revealed that the neural bias toward happiness or fearfulness differs as a function of the duration of EBF. Specifically, longer breastfeeding duration is associated with a happy bias, whereas shorter breastfeeding duration is associated with a fear bias. These findings suggest that breastfeeding experience can shape the way in which infants respond to emotional signals.

## Introduction

### Emotion discrimination in infancy

The ability to perceive and distinguish between the emotional states of others is a crucial social skill that helps us predict others’ actions and guide our own behavior during social interactions (Frith, 2009). Emotional communication is inherently multisensory, as information about another person’s emotional state can be gleaned from various channels (Heberlein and Atkinson, 2009). Most research has focused on the perception of emotions from facial and vocal expressions (Belin et al., 2013), even though body expressions may be the most evolutionarily preserved and immediate means of conveying emotional information (de Gelder, 2006; Aviezer et al., 2012). Adults are readily able to detect and recognize various emotions from body expressions (de Gelder, 2009; Atkinson, 2013). This ability to recognize emotions from body expressions relies on specific brain processes localized principally in the right hemisphere, including superior temporal, somatosensory and premotor cortices (Heberlein et al., 2004; Heberlein and Saxe, 2005; de Gelder, 2006; Grèzes et al., 2007; Atkinson, 2013).

In development, the ability to respond to emotional information emerges during the first year of life, during which time infants become sensitive to various facial, vocal, and body expressions (Grossmann, 2013; Missana et al., 2014a,b). There is behavioral and neural evidence to suggest that infants develop the ability to detect and discriminate between others’ positive and negative emotional expressions, tending to view the negative expressions as more salient (Vaish et al., 2008). For example, 7-month-old infants but not 5-month-old infants show longer looking times to fearful faces than to happy faces and an enhanced Negative central (Nc) component in their event-related brain potential (ERP), indexing greater allocation of processing and attentional resources to fearful expressions (Nelson and De Haan, 1996; Kotsoni et al., 2001; de Haan et al., 2003; Reynolds and Richards, 2005; Peltola et al., 2009). Similarly, 7-month-olds show an enhanced neural sensitivity to angry voices when compared to happy and neutral voices (Grossmann et al., 2005, 2010). Only recently has research begun to investigate the development of emotional body processing in infancy. Similar to what has been shown with facial and vocal stimuli, 8-month-old infants show an enhanced neural sensitivity to fearful relative to happy body expressions in both dynamic (Missana et al., 2014a) and static stimuli (Missana et al., 2014b).

Given this well-mapped emergence of the neural sensitivity to emotional information, it appears particularly important to understand which factors might contribute to individual differences during this early stage of development. To gain a better understanding of this might help inform theories about how certain biases in emotional processing come about. Such biases are thought to have long-term beneficial or detrimental effects on the well-being and development of an individual (i.e., negativity vs. positivity bias) (Vaish et al., 2008; Roiser et al., 2011; Sharot, 2011; Fox, 2012). In prior infant research, the focus of individual differences in the neural processing of emotions has mainly been on intrinsic factors such as genetic variation in neurotransmitter systems (Grossmann et al., 2011, 2013) and variation in temperament with respect to emotion regulation and expression (de Haan et al., 2004; Grossmann et al., 2011, 2013; Martinos et al., 2012). However, little is known about how certain experiential factors are linked to emotion perception and its neural underpinnings in infancy.

### Exclusive breastfeeding, development, and oxytocin

Prior research has recognized the general importance of maternal behavior and care in the early development and epigenetic programing of the mammalian offspring’s brain (Weaver et al., 2004; Cushing and Kramer, 2005; Masís-Calvo et al., 2013; Sarro et al., 2014). In human children, breastfeeding has been shown to play a prominent role in the promotion of healthy brain and cognitive development. For example, there is a wealth of longitudinal research suggesting that longer duration of exclusive breastfeeding (EBF)^1^ in infancy has beneficial effects on health in general but also on cognitive and intellectual development far into adult life (Mortensen et al., 2002; Oddy, 2002; Kramer et al., 2003; Daniels and Adair, 2005; Raju, 2011). Very recently, this has been qualified by the discovery that many neural outcomes, including total brain volume, cortical thickness, and white matter volume, are facilitated by the duration of EBF experience (Isaacs et al., 2010; Deoni et al., 2013; Kafouri et al., 2013).

While it is widely accepted that EBF is linked to improved cognitive development, most likely through its effects on neurodevelopment, very little is known regarding its potential impact on socio-emotional development (but see Hayatbakhsh et al., 2012; Peus et al., 2012). This is quite surprising, considering that breastfeeding is much more than simply a meal at the breast—it is generally considered to be a dynamic biological and psychological process that is fundamentally social in nature (Uvnäs-Moberg, 1998; Raju, 2011). Moreover, breastfeeding is intricately linked to the hormone oxytocin, which is required to stimulate the let-down of milk (Dawood et al., 1981). Oxytocin is also present in small quantities in human milk, and released during pleasant touch and warmth (Uvnäs-Moberg, 1998). Although cerebrospinal oxytocin levels in human infants have not been measured, animal research suggests that oxytocin is released in response to suckling (Lupoli et al., 2001). Oxytocin acts as a neuropeptide in the brain, and has been implicated in a wide variety of social processes and behaviors, particularly those related to affiliation and bonding (Carter et al., 1992; Young and Wang, 2004; Feldman, 2012b). There is accumulating evidence to suggest that oxytocin has specific effects on the perception of *positive* emotional expressions in adults (Guastella et al., 2008; Marsh et al., 2010; Gamer and Buchel, 2012; Domes et al., 2013). Nevertheless, the exact mechanisms through which oxytocin impacts emotion perception are still being debated (Bartz et al., 2011; Kemp and Guastella, 2011).

With respect to the link between EBF and emotion perception, a recent study by Krol et al. (2014) showed that the duration of EBF had a similar effect on positive emotion recognition in mothers as the intranasal administration of oxytocin in other groups of adults (Marsh et al., 2010; Domes et al., 2013). Specifically, Krol et al. (2014) were able to demonstrate that longer EBF predicted better (faster) recognition of happy facial expressions. This suggests that EBF is linked to improved processing and recognition of affiliative cues such as positive facial expressions and that this processing advantage may be due to increased oxytocin levels.

### The current study

From a developmental perspective, it is an open question whether EBF has an effect on emotion perception in infants and if so whether this effect is similar to what has been shown in mothers (Krol et al., 2014). Therefore, the current study sought to examine the effect of EBF duration on the neural processing of emotional information in 8-month-old infants. We chose to investigate the Nc ERP component, which has been used to indicate attention allocation in infants. Our rationale for using ERPs was that: (a) this method has been used repeatedly and reliably to elucidate emotion perception in nonverbal infants; and (b) ERPs and in particular the Nc have shown to be sensitive measures of individual differences in emotion perception in infancy (de Haan et al., 2004; Grossmann et al., 2011; Martinos et al., 2012). More specifically, the Nc is an electrophysiological correlate of infants’ allocation of attention and general orienting to a visual stimulus occurring around 400–800 ms post stimulus onset, most prominently observed as a negativity at frontal and central electrodes (Courchesne, 1977, 1978; Courchesne et al., 1981; Nelson, 1994; Nelson and Dukette, 1998; Nelson and Monk, 2001). There is evidence showing that under certain conditions the Nc is comprised of two peaks (Karrer et al., 1998; Ceponiene et al., 2004; de Haan, 2007)—an early peak occurring around 300–800 ms, and a later peak occurring from around 800 ms. To date, only one study has examined the neural underpinnings of emotion discrimination from static body expressions in infants. Missana et al. (2014b) found evidence for discrimination of emotion within the late Nc peak, but not within the early peak. We therefore chose to focus our analysis on the same time window (700–800 ms) as used in prior work. For this reason, we will refer to our specific time window as the “late Nc”.

As in prior work, we presented infants with happy and fearful body expressions in an upright and inverted orientation (Missana et al., 2014a,b). The inverted stimulus presentation was used as a control because body inversion has been shown to disrupt emotion recognition in adults (Atkinson et al., 2004) and emotion discrimination in infants (Missana et al., 2014a,b; Zieber et al., 2014). On the basis of prior work with oxytocin (Marsh et al., 2010; Domes et al., 2013), we predicted that the duration of EBF is associated with differences in positive (happy) emotion processing. Specifically, similar to what has been shown for mothers (Krol et al., 2014), we hypothesized that infants who were exclusively breastfed for a longer duration would show an increased neural sensitivity to positive (happy) expressions. This neural sensitivity would be manifested by a larger, more negative late Nc. Importantly, we assessed a number of other variables that might be linked to breastfeeding and emotion perception, such as infant temperament, maternal dispositional interpersonal reactivity (empathic concern (EC)), parity, and maternal education. Focusing our investigation on differences in the duration of EBF allowed us to examine variation within a group of breastfed infants, as opposed to comparing bottle-fed infants to breastfed infants.

## Methods

### Participants

The final sample included 28 infants (15 females) of European descent aged 243–261 days (*M* = 250.39, *SD* = 4.031) (about 8.23 months old), 13 of which whose data appear in an already published sample (Missana et al., 2014b). An additional 10 infants were tested, but were excluded from the final sample due to a lack of questionnaire data (*n* = 4), fussiness (*n* = 2), artifacts (*n* = 2), and experimenter error (*n* = 2). The infants were born full-term (between 37 and 41 weeks) and had a normal birth weight (>2500 g). Six infants were delivered via cesarean section and the rest underwent standard vaginal deliveries. All mothers that participated in our study had been on maternity leave up to the time of testing. All mothers provided informed consent prior to participation and were compensated with travel money and a toy for the infant. Procedures were approved by the ethics committee of the Leipzig University Medical School.

### Stimuli

**Figure 1:**
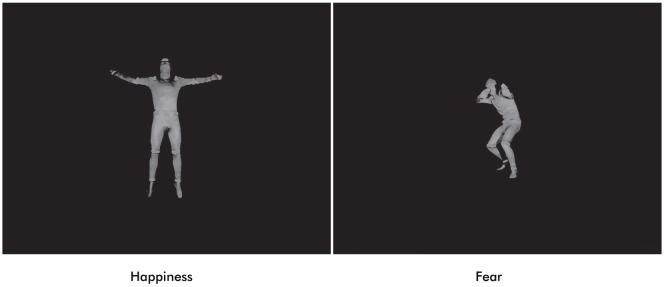
**Examples of full-light static body expressions of happiness and fear**.

Stimulus material consisted of full-light static body expressions portraying six fearful and six happy expressions in both upright and inverted orientations. Still frames of a previous dataset of dynamic body expressions were selected at the peak of emotional expression (Atkinson et al., 2004; Missana et al., 2014b; Figure 1). From the original set of eight stimuli per condition, six were chosen for each emotion on the basis of recognition rate by a group of adult raters, indicating at least 40% average correct identification of the displayed emotion (chance level was 16.7%).

### Procedure

Infants were seated on their mothers’ laps in a dimly lit, electrically shielded, and sound-attenuated room during stimulus presentation. Mothers were instructed to focus their attention on the child and to ignore the presented stimuli. Stimuli were displayed on a 70 Hz, 17-inch computer screen with a distance of about 70 cm. Images were presented in the center of the screen with a black background. Each image was preceded by an alerting sound in order to attract infants’ attention. Each presentation trial began with a fixation cross (1000 ms) followed by a black screen (400 ms), followed by the stimulus for 2000 ms. An abstract screensaver was presented during the inter-stimulus interval in order to obtain the infants’ attention for the subsequent trial. Sessions were video recorded online in order to ensure infants were looking at the screen. Trials were presented manually by button press. Stimuli were presented in a randomized order with the exception that no two stimuli of the same emotion/orientation combination were presented consecutively. The EEG session ended when the infant became fussy or inattentive. An average of 15.53 trials were presented per condition.

### ERP analysis

EEG was recorded from 27 Ag/AgCl electrodes attached to an elastic cap designed for infants using the 10–20 system of electrode placement (EasyCap GmbH, Germany). Electrophysiological data was online referenced to the central CZ electrode and re-referenced offline to the average of the left and right mastoid electrodes. The horizontal electrooculogram (EOG) was taken from two electrodes (F9 and F10) located on the outer canthus of each eye. Vertical EOG was recorded from an electrode on the supraorbital ridge (Fp2) and an additional electrode placed on the infraorbital ridge of the right eye. EEG was amplified using a Porti-32/M-REFA amplifier (Twente Medical Systems International) and digitized at a rate of 500 Hz. Impedences were kept between 5 and 20 kΩ. Data processing for subsequent ERP analysis was performed with an in-house software package EEP, commercially available as EEProbeTM (Advanced Neuro Technology, Netherlands). Raw EEG data was bandpass filtered between 0.3 and 20 Hz. Recordings were segmented into epochs which were time-locked to the stimulus onset, lasting from 200 ms before the onset until the offset of each stimulus (a 2200 ms duration). Epochs were baseline-corrected by subtracting the average voltage of the 200 ms baseline period prior to image onset. Epochs were rejected offline if the standard deviation within a gliding window of 200 ms exceeded 80 µV in any two of the bipolar EOG channels and 60 µV at EEG electrodes. Artifact-free epochs were averaged at each electrode for each condition (happy upright, happy inverted, fearful upright, and fearful inverted) in order to acquire ERPs. Each infant contributed an average of 5.5 artifact-free trials per condition (the average trial number did not differ between EBF groups: low EBF: 5.9, high EBF: 5.2). In a recent infant ERP methods review, Hoehl and Wahl (2012) demonstrate that five trials are sufficient to evoke clear Nc responses in infants using similar visual stimuli and working with infants of a very similar age as in our study.

**Figure 2:**
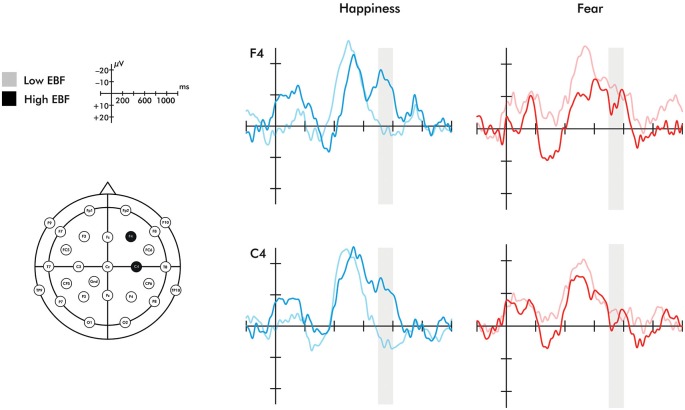
**Event-related potentials for the F4 and C4 electrodes in response to happiness and fear**. Region shaded in gray represents the time window of the Nc analyzed. Please note that the time windows of 0–200 ms and 400–600 ms did not differ significantly between groups for either electrode.

A time window of 700–800 ms was chosen to investigate the late ERP negative central component (Nc) in the right hemisphere. The selection of the time window was based on prior work (Missana et al., 2014b) showing that Nc effects of emotional body expressions are seen later than in prior ERP work using emotional facial expressions. The selected time window still falls within the range of the commonly studied time range for investigating effects on the Nc (400–800 ms) (Courchesne et al., 1981). As stated in the introduction, some studies have found a two-peaked Nc (Karrer et al., 1998; Ceponiene et al., 2004). The ERP waveforms presented in Figure 2 indicate that the Nc is comprised of two peaks in the longer duration of EBF group. Mean amplitude effects were extracted from two ROIs: right fronto-central electrodes (F4, C4) and left fronto-central electrodes (F3, C3).

### Breastfeeding questionnaire and analysis

A 10-item breastfeeding questionnaire was created in order to obtain demographic information as well as the following measures: the amount of time a mother *exclusively* breastfed her child (providing her child breast milk from the breast as the only source of sustenance), at what age the child was first introduced to other foods (if at all), and/or at what age the mother stopped breastfeeding the child. Whether the mother had breastfed in the last 24 h was also recorded. The questionnaire provided a table in which mothers could describe their infant’s feeding schedule (what time of day and what food intake) over the course of a normal day. Through this, frequency of breastfed meals per day could be calculated, as well as the percentage of daily meals that were breastfed. Additional information such as the age and parity of the mother, education, job, and immigration history was also collected. Durations provided in months (i.e., 6 months of EBF) were converted into days. If mothers were still exclusively breastfeeding, the infants’ age on testing day was used in order to be as specific as possible. The duration of EBF was normally distributed in our sample and showed no significant skewness or kurtosis, *M* = 150.55 days, *SD* = 65.18. A mean split was obtained from EBF duration in order to create categorical groups of low and high EBF for further analysis and visualization (low EBF: *M* = 102.66 days, *SD* = 53.21; high EBF: *M* = 198.43 days, *SD* = 32.45).

### Additional questionnaires

The revised Infant Behavior Questionnaire (IBQ-R; Gartstein and Rothbart, 2003) was used to assess general infant temperament. This questionnaire is known internationally for its ability to gain insight into the unique temperaments of infants, including subscales assessing infant expression of smiling and laughter, approach tendency, perceptual sensitivity, cuddliness, fear, rate of recovery from distress, and many others. Additionally, the Interpersonal Reactivity Index (IRI; Davis, 1983) was given to mothers to assess dispositional empathy. This questionnaire includes four subscales, assessing the tendency one has to take the point of view of others (perspective taking (PT)), the tendency to experience feelings of sympathy and compassion for those less fortunate (EC), the tendency to experience feelings of distress in response to discomfort in others (personal distress (PD)), and the tendency to transpose oneself into fictional situations such as novels or movies (fantasy seeking (FS)).

## Results

The breastfeeding characteristics of the sample are presented in Table 1. Out of our 28 infants, 14 were still breastfed at least one meal a day. There were no EBF group differences in birth weight or gestation duration (*F*_(1,26)_ = 0.438, *p* = 0.514, *η*^2^ = 0.017; *F*_(1,26)_ = 0.122, *p* = 0.729, *η*^2^ = 0.005, respectively). Nor were there differences in maternal education or parity (*F*_(1,26)_ = 0.056, *p* = 0.816, *η*^2^ = 0.002; *F*_(1,26)_ = 0.140, *p* = 0.712, *η*^2^ = 0.005). EBF duration was not significantly impacted by delivery method (cesarean section or vaginal delivery) (*F*_(1,25)_ = 0.177, *p* = 0.678, *η*^2^ < 0.001). Furthermore, there were no EBF group differences across any of the IBQ-R infant temperament subscales or maternal dispositional empathy as assessed through the IRI (all *p*-values > 0.05). Note that correlations with EBF as a continuous variable did not reveal any significant covariates (all *p*-values > 0.05). However, there is some evidence that maternal education might be a predictor of EBF duration (DiSantis et al., 2013). We therefore included years of maternal education as well as current breastfeeding exposure (percent of breastfed meals/day) as covariates in all subsequent analyses.

**Table 1:** **Breastfeeding descriptives**.

	EBF low (*N* = 14)		EBF high (*N* = 14)			Total (*N* = 28)	
	*Range*	*M(SD)*	*Range*	*M(SD)*	*p*-value	*Range*	*M(SD)*
EBF duration (days)	12–152	102.66 (53.2)	167–253	198.43 (32.5)	<0.001	12–253	150.55 (65.2)
Breastfed meals/day (%)	0–50	7.20 (15.4)	0–87.50	35.31 (28.6)	0.003	0–87.50	21.25 (26.7)
Other children (#)	0–1	0.43 (0.5)	0–1	0.36 (0.5)	*ns*	0–1	0.39 (0.5)
C-sections (#)	*na*	0.21 (0.4)	*na*	0.23 (0.4)	*ns*	*na*	0.22 (0.4)
Education (years)	13–22	17.12 (3.2)	13–22	16.86 (2.4)	*ns*	13–22	16.98 (2.8)
Maternal age	26–35	30.93 (3.2)	26–37	31.51 (3.5)	*ns*	26–37	31.22 (3.3)

**Figure 3:**
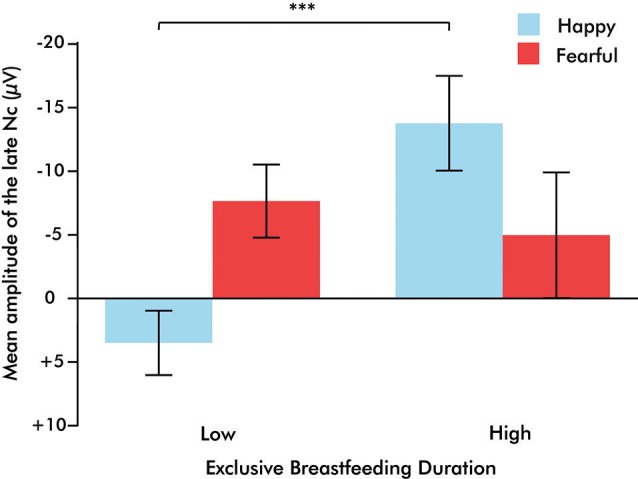
**Bar graph illustrating the emotion × exclusive breastfeeding experience (EBF) interaction, in which infants with high EBF duration demonstrate a greater Nc to happiness than to fear, and infants with low EBF duration demonstrate a greater Nc to fear than to happiness**. Bars represent standard error of the mean, ***p = 0.001.

A 2 (emotion: fearful, happy) × 2 (orientation: upright, inverted) × 2 (EBF experience: high, low) repeated measures ANOVA was conducted on the averaged right fronto-central (F4 and C4) late Nc. This analysis revealed a significant emotion × orientation × EBF experience interaction (*F*_(1,20)_ = 7.134, *p* = 0.015, *η*^2^ = 0.263). To explore this three-way interaction further, 2 (emotion: fearful, happy) × 2 (EBF experience: high, low) repeated measures ANOVAs were conducted on the late Nc for upright or inverted expressions separately. This analysis yielded a highly significant emotion × EBF interaction in the upright condition (*F*_(1,24)_ = 14.444, *p* = 0.001, *η*^2^ = 0.376; Figures 2, 3). Infants in the low EBF group showed greater (more negative) late Nc responses to fearful expressions than to happy expressions, suggesting a greater allocation of attention to fearful stimuli. In contrast, high EBF infants displayed greater late Nc responses to happy expressions and more positive late Nc responses to fearful expressions, suggesting greater attention towards happy stimuli. Further exploration of this interaction revealed that group differences in emotional processing were driven specifically by the late Nc to *happiness*, as group averages did not differ significantly for fear (happiness: *F*_(1,26)_ = 14.667, *p* = 0.001, *η*^2^ = 0.361, fear: *F*_(1,26)_ = 0.220, *p* = 0.643, *η*^2^ = 0.008 (difference for happiness survives Bonferroni correction at *p* < 0.025). Critically, there was no interaction between emotion and EBF experience in the inverted condition, *F*_(1,20)_ = 4.487, *p* = 0.237, *η*^2^ = 0.069, suggesting that the interaction effect is specific to emotional stimuli presented in an upright orientation. Note that, in line with prior work showing that the detection and discrimination of emotional body expressions is more prominent over the right hemisphere (Missana et al., 2014a,b), no emotion × orientation × EBF duration interaction was observed for frontal and central electrodes (F3 and C3) over the left hemisphere (*F*_(1,20)_ = 3.457, *p* = 0.078, *η*^2^ = 0.147).

**Figure 4:**
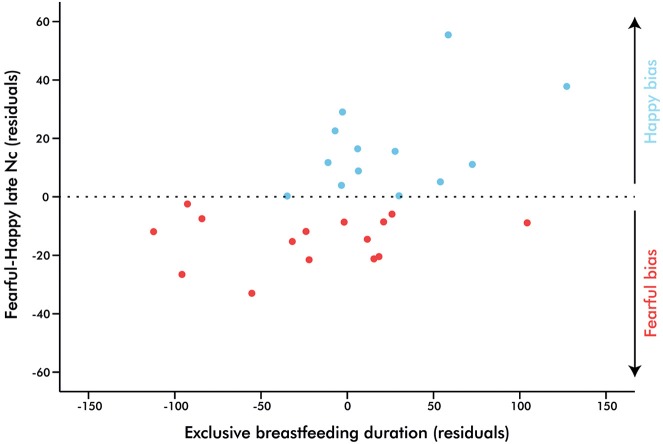
**Partial regression plot illustrating the prediction of exclusive breastfeeding duration on infant brain response to fearful-happy expressions**. Points above zero represent a bias towards happy stimuli, while points below zero represent a bias towards fear as indexed by the right fronto-central Nc (700–800 ms), *p* = 0.01.

**Figure 5:**
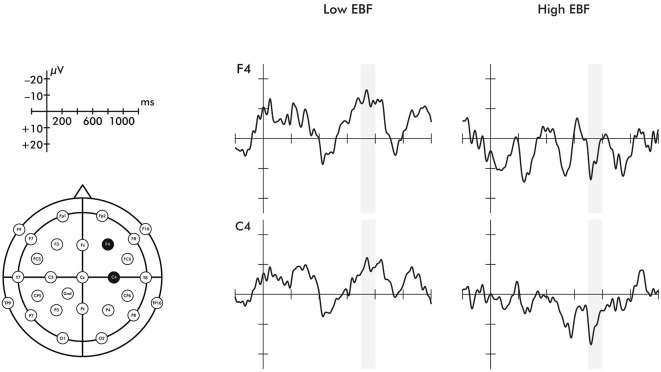
**Event-related potentials for the F4 and C4 electrodes representing the response to fearful minus happy stimuli**. Region shaded in gray represents the time window of the Nc analyzed. These are shown by exclusive breastfeeding duration (EBF) group for illustrative purposes, but note that the difference score was treated as a continuous variable in analyses.

Additionally, a difference score was computed in order to investigate a potential linear relationship between emotional biases of the late Nc amplitude with the duration of EBF. A forced-entry regression model was conducted in which EBF duration, maternal education, and current breastfeeding exposure predicted the fearful-happy Nc. EBF duration was the only significant predictor, suggesting that as days of EBF increase, the attentional bias towards fear shifts towards one for happiness (Table 2, Figure 4). Note that the impact of EBF on the fearful-happy Nc survives Bonferonni correction at *p* < 0.0167. The difference score waveforms are shown in Figure 5, separated by EBF group for visualization purposes.

**Table 2:** **Multiple regression indicating a positive prediction of EBF duration on the Nc in response to fearful expressions minus Nc in response to happy expressions**.

	B	SE B	β	*t*	*p*-value
EBF duration (days)	−0.17	0.06	−0.56	−2.75	0.01
Breastfed meals/day (%)	0.22	0.16	0.29	1.37	0.19
Education (years)	0.44	1.35	0.06	0.32	0.75

*Note: R^2^: 0.244*.

## Discussion

Our results revealed that EBF duration was linked to differences in the neural processing of emotional body expressions in 8-month-old infants. Specifically, infants with a high duration of EBF showed a significantly greater late Nc component to happy body expressions than those with a short duration of EBF, suggesting a greater allocation of attention to happiness with extended EBF. This demonstrates that prolonged EBF in infants is associated with an increased sensitivity to positive emotional information in a similar fashion as shown in prior work with mothers (Krol et al., 2014). Such an increased sensitivity to positive emotional signals in both infants and mothers may be important in fostering positive social interactions and thereby might serve important affiliative and bonding functions in human development (Feldman, 2012a). Moreover, our analysis revealed that the bias toward positive or negative expressions shifted in a linear fashion as a function of EBF. Infants who were exclusively breastfed for a longer duration (above 152 days (approximately 5 months)) showed a positivity bias in their ERP responses (greater late Nc in response to happy than to fearful expressions), while infants who were exclusively breastfed for a shorter duration (below 152 days) showed a negativity bias in their ERP responses (greater late Nc in response to fearful than to happy expressions). This is the first evidence to suggest that emotion processing in infancy critically differs as a function of breastfeeding experience, supporting the notion that breastfeeding behavior is a complex biological and psychological process linked to early socio-emotional development.

Importantly, our results showed that the association between the duration of EBF and emotion processing in infancy exists independent of other variables that might have impacted the duration of EBF. In particular, we ruled out that EBF is linked to any of the following variables in our sample: several aspects of infant temperament (i.e., smiling and laughter, fear), maternal dispositional interpersonal reactivity (i.e., EC), and parity. Current breastfeeding exposure (the percent of breastfed meals per day) and maternal education (in years) were factored into every analysis to further validate specific results of EBF. Furthermore, the association with EBF was not observed when the emotional stimuli were presented upside-down, which has been shown to disrupt emotion discrimination and recognition (Missana et al., 2014a,b), but was specific to emotional body expressions presented in the upright orientation. This strengthens our findings by showing that the association with EBF is specific to when emotional information can be detected in the stimulus.

An important point for discussion is related to the finding that the ERP differences between EBF groups were evident for a relatively late time window of the Nc. Specifically, infants in the longer duration of EBF group showed an additional negative deflection (peak) that followed the main Nc. Interestingly, there is prior work showing that under certain conditions the Nc comprises two peaks (Karrer et al., 1998; Ceponiene et al., 2004; de Haan, 2007). Specifically, while the first peak has been linked to initial attentional orienting, the later second peak is viewed as reflecting sustained attention, possibly as a function of the salience of the stimulus. This difference in the timing of these processes might also map onto distinct cortical sources as an early aspect of the Nc is generated in anterior cingulate cortex (ACC) and medial frontal gyrus and a later aspect of the Nc is localized to the frontal pole (Reynolds and Richards, 2005). With respect to the current findings, this may indicate that infants with high EBF experience recruit additional brain processes related to sustained attention when viewing happy body expressions, reflecting the salience of positive expressions for this group. Future work using neuroimaging techniques that allow for the precise mapping of cortical activation, such as functional near-infrared spectroscopy (fNIRS), is needed to further examine the possibility that infants recruit such specific brain processes linked to the frontal pole under these conditions (see Grossmann et al., 2008; Grossmann and Johnson, 2010 for infant fNIRS work imaging prefrontal cortex including frontal pole).

A further point for discussion relates to the role that oxytocin may play in the development of the emotional processing biases described in the current study. Our finding of an increased sensitivity to positive emotional expressions in infants with prolonged EBF is not only in line with what has been found in mothers (Krol et al., 2014) but also fits well with what has been shown with intranasal administration of oxytocin (Marsh et al., 2010; Domes et al., 2013). Furthermore, animal research has shown that breastfeeding increases central oxytocin levels in both mother and offspring (Dawood et al., 1981; Lupoli et al., 2001) and is thought to have similar effects in humans (Uvnäs-Moberg, 1998). It is also important to note that genetic differences in the oxytocin system have been found to give rise to morphometric alterations in healthy human adults particularly in limbic regions including the hypothalamus, amygdala, and ACC (Tost et al., 2010). As the infant Nc is believed to arise from frontal cortical regions, and in particular, the ACC (Reynolds and Richards, 2005), this could point to a method in which chronic modulation of the oxytocin system might impact structural differences in the brain which mediate attention and emotional processing. Given the converging picture from the current study and prior work, we would like to tentatively suggest that EBF affects central oxytocin levels in infants and thereby impacts emotion processing. In future work, it is crucial that this proposal be tested more directly by obtaining genetic and physiological information regarding the oxytocin system.

Regardless of the exact mechanism by which breastfeeding contributes to individual differences in emotion processing, there is also a need to discuss the current findings with respect to the general role that breastfeeding behavior may play in socio-emotional functioning. More specifically, the current findings have shown that infants’ responsiveness to emotional information varies as a function of their breastfeeding experience and that biases towards positive and negative information are linked to the duration of EBF. Prior work demonstrates that, on average, infants begin to display a negativity bias around 7 months of age, particularly seen as an increased attention and neural sensitivity to fearful facial expressions (Vaish et al., 2008). Our results suggest that this general developmental trajectory might be modulated by EBF experience. Interestingly, the neural response to happy body expressions elicited in the high EBF group appear similar to that of the average neural response to fearful body expressions found by Missana et al. (2014b) in prior work as well as the infants in the low breastfeeding group. While this suggests that infants’ allocation of attention to emotional stimuli may differ depending on their breastfeeding experience, we would like to caution against the interpretation that infants in the high breastfeeding group somehow perceive happy expressions as fearful or threatening. This is because the neural index used in this study (late Nc) simply provides a correlate of attention allocation but does not tell us about the emotional valence of the perceived stimulus.

To understand why it is that some infants show what has been referred to as a negativity bias, while others show a positivity bias can help inform accounts of socio-emotional development by providing insights into the experiential dependence of emotional functioning (see Vaish et al., 2008). There are at least two possibilities to functionally interpret the current findings. First, the duration of EBF and the (assumed) exposure of the infant to increased levels of oxytocin may, during this sensitive phase of emotional development, have an early programing effect. Infants who are exclusively breastfed longer may become more sensitive to positive information, while infants who are exclusively breastfed for shorter durations may become more sensitive to negative information. This view is in agreement with work showing that maternal behavior can program stable changes in gene expression and neurobiological functioning that provide the basis for individual differences in socio-emotional behavior of the offspring (e.g., Kappeler and Meaney, 2010). Second, the cessation of EBF is often linked to the mother’s effort to wean a child. Therefore, it is possible that the impact of EBF duration on emotion processing may reflect a consequence of late weaning. In this scenario, the later weaned infant may rely more on the mother, rendering negative or threatening information less important to attend to. Relatedly, earlier weaning may engender processes that sensitize the infant to negative information, as weaning is a first correlate of more independent functioning. This may thus be reflected in the way negative information is approached. Importantly, these two frameworks for explaining the current findings make rather different but testable developmental predictions. According to the programing account, infants that show a negativity or positivity bias at 8 months should still show the same kind of bias during later stages of development. In contrast, according to the late weaning account, infants that show a positivity bias at 8 months would also start showing a negativity bias once they are weaned. It is thus of great importance to investigate the impact of EBF on socio-emotional functioning across development in longitudinal studies. Animal research will also help to explore how maternal nursing behavior impacts the neural and social development of their young. For example, recent work by Sarro et al. (2014) reports that nipple attachment and milk ejection impact the cortical synchrony of rat pups. These novel findings suggest possible mechanisms through which prolonged maternal care might induce robust individual differences in neural maturation.

While the current findings speak to the impact that maternal behavior has on infant social brain function, it is important to note that infants showed differential effects on processing emotions in response to (female) strangers. This suggests that the breastfeeding experience with the mother, while certainly having specific effects on the relationship between infant and mother, is also linked to more general processing differences that can be observed in response to people’s body expressions that are unfamiliar to the infant. To find that specific social experiences, especially early in development, can have general effects on social responsiveness, is in line with a host of work examining individual differences in early social development (Pollak and Kistler, 2002; Pollak and Sinha, 2002; Fries et al., 2005; Curtis and Cicchetti, 2013).

Even though we took great care in ascertaining the specific influence of EBF on emotion processing, it should be acknowledged that other factors that were not examined in the current study may be linked to EBF and/or emotion perception and may thus influence the effects. For example, co-sleeping impacts how often the infant is able to breastfeed at night (Buswell and Spatz, 2007), and early sleep patterns in general may influence later social development (Dahl, 1996). We also did not investigate maternal mood or history of postpartum depression. Analyses from another cohort of mothers (Krol et al., 2014) found no correlation of either positive or negative mood on EBF duration. However, it could be possible that the maternal mood state or physical contact impact infants’ perception of emotional expressions. It is also very possible that infant temperament might not only impact a mother’s decision to continue or cease breastfeeding, but also might impact the infant’s perception of emotional expressions. However likely, we did not find any influence of infant temperament in the current sample. Another point is that parental gesture behavior might influence how familiar infants are with emotional body expressions. Future studies might benefit from recording natural interactions between the mother and infant, such that data regarding physical touch, gesturing, and affect can be taken into account. Moreover, due to the nature of our hypothesis (exploring variation within a group of breastfed infants), our study lacks a control group. One suggestion for future research is to include a group of exclusively formula-fed infants matched for the time they were introduced to solid foods. Such a control would help to parcel out specific influences of breastfeeding.

Finally, it must be stressed that although we discuss the role oxytocin may play in the processes studied here, we cannot confirm that EBF duration and oxytocin levels are correlated in our sample. Indeed, this has yet to be confirmed in any sample of human infants, and we must rely on animal research for the time being (Lupoli et al., 2001). It must also be acknowledged that the act of breastfeeding is a dynamic activity, which impacts several hormonal systems, physiological states, and brain processes. For example, breastfeeding reduces hypothalamic-pituitary-adrenal (HPA) axis activity in mothers (Heinrichs et al., 2002) as well as increases activation of several limbic regions in the maternal brain (Kim et al., 2011). Moreover, breastfeeding involves skin-to-skin contact and pleasant touch, which has been found to influence emotion regulation, attention, joint engagement, and pain analgesia (Gray et al., 2000; Feldman et al., 2014), as well as reducing heart rate responses in infants (Fairhurst et al., 2014). Until directly tested, increased oxytocin exposure must only be considered a potential pathway (among others) that can account for our current results. Future work will benefit from the addition of hormonal and genetic markers in both mothers and infants in order to achieve a more detailed and possibly more mechanistic understanding of the effects of EBF on socio-emotional processing.

In conclusion, the current study provides first insights into the role that EBF plays in contributing to individual differences during the neural processing of emotional expressions in infancy. Our results demonstrate that infants who had been exclusively breastfed longer showed an increased neural sensitivity to positive (happy) body expressions, while infants who had less EBF experience showed an increased neural sensitivity to negative (fearful) body expressions. The finding that such biases in emotional information processing occur in the context of the psychological and biological processes associated with breastfeeding during infancy is testament for a need to better understand the impact that maternal care in general and breastfeeding in particular has on socio-emotional functioning in infancy.

## Conflict of interest statement

The authors declare that the research was conducted in the absence of any commercial or financial relationships that could be construed as a potential conflict of interest.

## Footnotes

^1^EBF refers to the amount of time an infant was fed with breast milk (from the breast and not from a bottle) as the *only* source of sustenance; e.g., EBF stops when the child is introduced to solid foods (Kramer et al., 2003).
